# Who Needs Microtubules? Myogenic Reorganization of MTOC, Golgi Complex and ER Exit Sites Persists Despite Lack of Normal Microtubule Tracks

**DOI:** 10.1371/journal.pone.0029057

**Published:** 2011-12-27

**Authors:** Kristien J. M. Zaal, Ericka Reid, Kambiz Mousavi, Tan Zhang, Amisha Mehta, Elisabeth Bugnard, Vittorio Sartorelli, Evelyn Ralston

**Affiliations:** 1 Light Imaging Section, Office of Science and Technology, National Institute of Arthritis, Musculoskeletal, and Skin Disease, National Institutes of Health, Bethesda, Maryland, United States of America; 2 Laboratory of Muscle Stem Cells and Gene Regulation, National Institute of Arthritis, Musculoskeletal, and Skin Diseases, National Institutes of Health, Bethesda, Maryland, United States of America; 3 Institut Curie, Centre Universitaire, Orsay, France; University of Colorado, Boulder, United States of America

## Abstract

A wave of structural reorganization involving centrosomes, microtubules, Golgi complex and ER exit sites takes place early during skeletal muscle differentiation and completely remodels the secretory pathway. The mechanism of these changes and their functional implications are still poorly understood, in large part because all changes occur seemingly simultaneously. In an effort to uncouple the reorganizations, we have used taxol, nocodazole, and the specific GSK3-β inhibitor DW12, to disrupt the dynamic microtubule network of differentiating cultures of the mouse skeletal muscle cell line C2. Despite strong effects on microtubules, cell shape and cell fusion, none of the treatments prevented early differentiation. Redistribution of centrosomal proteins, conditional on differentiation, was in fact increased by taxol and nocodazole and normal in DW12. Redistributions of Golgi complex and ER exit sites were incomplete but remained tightly linked under all circumstances, and conditional on centrosomal reorganization. We were therefore able to uncouple microtubule reorganization from the other events and to determine that centrosomal proteins lead the reorganization hierarchy. In addition, we have gained new insight into structural and functional aspects of the reorganization of microtubule nucleation during myogenesis.

## Introduction

The Golgi complex is traditionally thought of as a single organelle per cell, and represented as a stack of flattened cisternae next to the nucleus. However, alternative organizations are found in skeletal [1], [2], [3] and cardiac [4] muscle, in osteoclasts [5], [6], plant cells [7], yeasts [8], polarized endothelial cells [9] and Drosophila embryos [10], [11]. The Golgi complex organization is also altered during mitosis [12], [13], [14], in apoptotic cells [15], in diseases such as Amyotrophic Lateral Sclerosis [16], [17], and in animal models of diseases such as Duchenne Muscular Dystrophy [18], [19], [20] and Pompe Disease [21], [22], [23]. Understanding how the Golgi complex transitions between different morphologies should help us assess the consequences of these modifications.

Skeletal muscle cell cultures are particularly informative since their Golgi complex transitions from a classic to an alternative fragmented organization during differentiation. This reorganization coincides with changes of the microtubule-organizing center (MTOC), from a classic centrosome to a combination of perinuclear belt and centrosomal remnants, and with remodeling of the microtubule network [1], [2], [24]. The reorganization of the Golgi complex also coincides with that of the ER exit sites (ERES) [1], [2], [24], [25]. The muscle Golgi complex continues to be remodeled during *in vivo* myogenesis to form a fiber type-dependent network of hundreds of small stacks of cisternae, closely associated with ERES and positioned throughout the fibers [3], [26]. These small Golgi complex elements are associated with a three-dimensional microtubule lattice [26], [27].

Reciprocal relations between the MTOC, microtubules, Golgi and ERES make the matter more complicated: centrosomes nucleate microtubules but, conversely, microtubules are involved in keeping the essential centrosomal proteins pericentrin and γ-tubulin in the centrosome [28], [29]. Similarly, microtubules are necessary for the integrity of the Golgi complex [30], which is positioned near the centrosome by minus-end directed microtubule motors [31]. However, it is now accepted that the Golgi complex itself is involved in microtubule nucleation [32], [33]. Finally, ERES themselves are positioned along microtubules through interaction of their COPII coat proteins with dynactin [34]. Golgi complex elements in differentiated muscle cells can thus interact with microtubules directly, or indirectly through the ERES. In light of all these potential interdependencies, the hierarchy of the microtubule-Golgi complex changes during muscle differentiation is far from clear.

Previously we have shown similarities between Golgi complex changes during myogenesis and during microtubule depolymerization [24], [25]: in both cases, the Golgi complex becomes fragmented, and the resulting elements are positioned at ERES. However, Golgi fragments produced by microtubule depolymerization do not form a perinuclear belt, but are dispersed through the cytoplasm [35]. These results suggested that microtubules might be dispensable for some but not all steps of Golgi complex reorganization during myogenesis. To test this notion and to clarify the interdependence of the several reorganizations (MTOC, microtubules, Golgi complex, ERES), we decided to uncouple them by using microtubule-altering drugs.

The results presented here show that altering or removing microtubules does not prevent myogenic reorganizations. Centrosomal proteins provide the platform for the positioning of the Golgi complex and ERES at the nuclear envelope and emerge as the key players. We also present evidence that the reorganizations proceed by progressive modification of existing structures rather than by demolition and *de novo* rebuilding.

Finally, we used EB3-GFP to compare microtubule nucleation in myoblasts and myotubes, at steady-state and after microtubule depolymerization. These experiments show that nucleation occurs constantly from the nuclear membrane of myotubes. They also reveal differences between centrosome and nuclear membrane as to MTOC organization.

## Results

### Centrosome, Golgi complex and ERES reorganizations resist microtubule alterations

In order to assess the participation of microtubules in the reorganization of MTOC, Golgi complex and ERES during muscle development, we challenged cultures of the mouse skeletal muscle cell line C2 [36] to differentiate in the presence of microtubule-altering drugs. Besides taxol and nocodazole, whose cell-wide microtubule stabilization and destabilization, respectively, are well known, we also used a specific inhibitor of GSK3-β, DW12 [37]. Inhibition of GSK3-β affects cell migration in the wound model by preventing the preferential stabilization of microtubules in the direction of cell migration [38], [39], [40]. We have found that DW12 (like lithium chloride, a much less specific inhibitor of GSK3-β) stabilizes microtubules preferentially around the nuclei.

Our aim was to disturb the microtubule network before differentiation starts in the unsynchronized C2 cultures, while keeping cells differentiation-competent. Therefore, the drugs were added 4 hours after plating the cells in growth media (GM). For each drug, we tested a range of concentrations. We found that 50 ng/ml of nocodazole sufficed to stop cell growth (Figure S1A), but we used 200 ng/ml, the minimum concentration needed to remove all large microtubules in 48 hours. A similar test for taxol and DW12 concentrations led us to choose 50 nM and 100 nM respectively. After 48 hours in GM in the presence of the drugs, the desired effects on microtubules were indeed achieved (Figure S1B). Myogenin, the marker used to assess differentiation, was expressed in fewer than 15% of the cells (Figure S1C). Differentiation was initiated by switching the cultures to fusion medium (FM), and the effects of the treatments were assessed 24 hours later. By that time, control cultures' expression of myogenin had increased and small myotubes were present, indicating that differentiation was well under way.

Figure S2 shows the state of the microtubules at the end of the treatments. Glu-tubulin, an indicator of microtubule stabilization, was present with different patterns in taxol and DW12-treated cells but also in very short microtubules left in nocodazole-treated cells. We did not consider the inhibition of fusion by nocodazole and taxol (Figure S2) a hindrance, since the events we are monitoring precede fusion [24], [41]. All quantitations were done on myocytes (mononucleated, differentiated cells).

**Figure 1:**
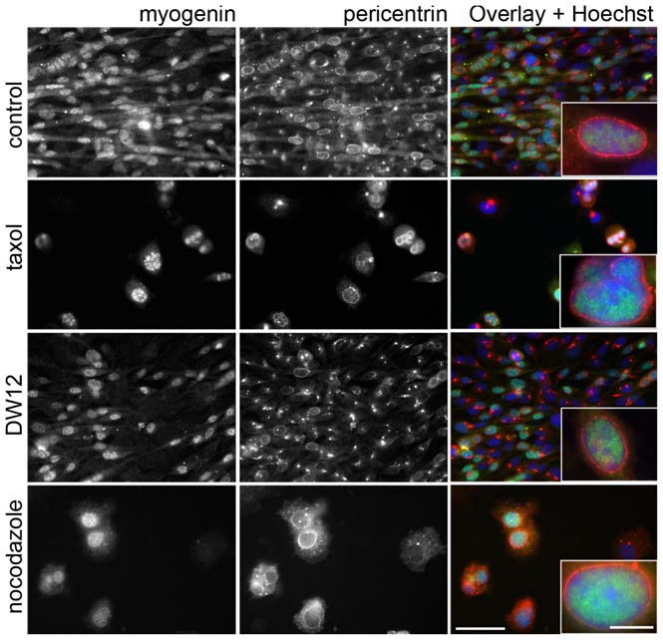
C2 cells initiate differentiation and reorganize pericentrin despite chronic treatment with microtubule-disrupting drugs. Cells differentiated in the presence of 50 nM taxol, 100 nM DW12 or 200 ng/ml nocodazole (see Methods) were stained for myogenin (green), a differentiation marker, and pericentrin (red), a centrosomal protein. Myogenin-positive cells show characteristic perinuclear pericentrin belts (insets) despite incomplete elongation, inhibition of fusion and enlarged nuclei (taxol and nocodazole). Wide-field images. Bars: 25 µm and 5 µm (insets).

**Figure 2:**
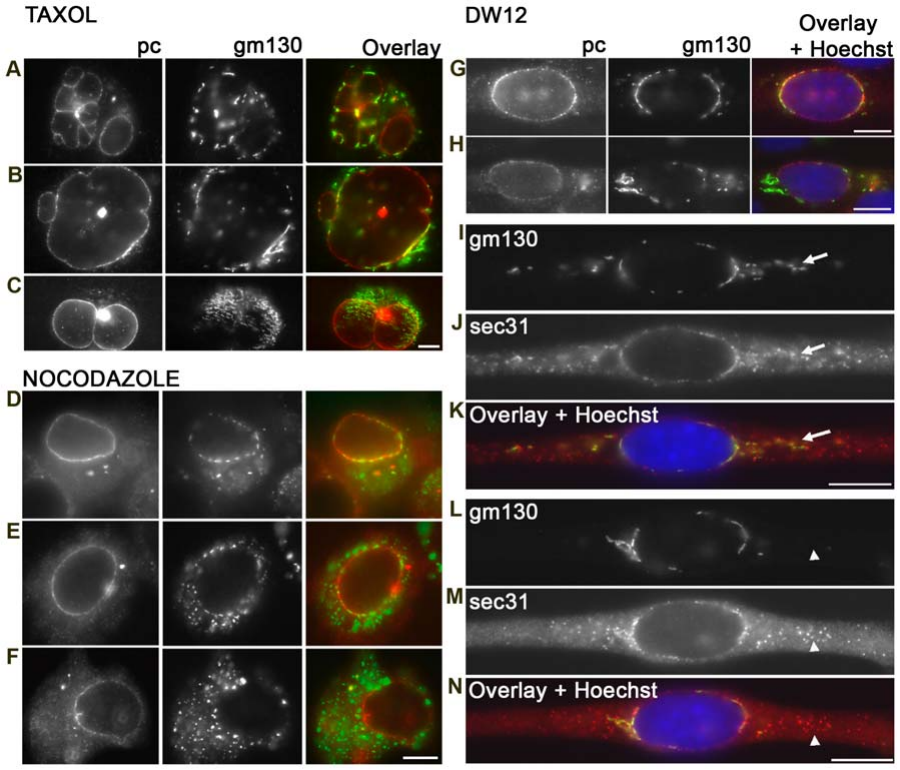
Reorganization of Golgi complex and ERES also takes place in cells treated with microtubule-altering drugs. (A–H) C2 cultures treated as described in Figure 1 were stained for pericentrin (pc, red) and GM130 (green) or (I–N) for GM130 (green) and Sec31 (red), an ERES marker. Only cells with redistributed pericentrin are shown here. In the absence of pericentrin redistribution there is no redistribution of GM130 or Sec31 (not shown here). In taxol- and nocodazole-treated cells, we find different degrees of Golgi complex reorganization: full (A, D); partial (B, E); none (C, F). In DW12 (G–N), Golgi fragment localization is polarized (G, H) or fragmentation is incomplete (H). Most ERES are associated with Golgi complex elements regardless of treatments (I–K; arrows). Rare cytosolic ERES clusters without Golgi elements are observed in the cytoplasm (L–N, arrowheads). Wide-field images. Bars: 20 µm.

We assessed the treatment effects by double-staining for myogenin versus pericentrin, GM130, or Sec31, to probe for reorganization of centrosome, Golgi complex, and ERES respectively and distinguish differentiated from undifferentiated cells. Results for pericentrin are shown in Figure 1. All cultures, regardless of treatment, showed increased myogenin expression. Furthermore, a large fraction of the myogenin-positive cells showed the perinuclear pericentrin staining typical of normal differentiation, even when cell and nuclear shape were abnormal. In contrast, none of the myogenin-negative cells showed reorganized pericentrin. Similarly, the Golgi complex and ERES were redistributed to the nuclear envelope of many of the myogenin-positive myocytes, regardless of drug treatment. Quantitation of these observations (Table 1) confirms that a normal microtubule (re)organization is not necessary for that of the other systems. In fact, pericentrin reorganization appeared specifically enhanced by taxol and nocodazole. Thus, pericentrin, Golgi complex and ERES may follow different pathways when reorganizing. We observed GM130 reorganization only in cells with reorganized pericentrin (Figures 2 and S3), thus pericentrin reorganization appears upstream of the Golgi complex reorganization.

**Table 1:** Quantitation of redistribution of pericentrin, Golgi complex and ERES in myogenin-positive cells after differentiation in microtubule-altering drugs.

	control	taxol	nocodazole	DW12
Perinuclear pericentrin	48±7	76±12*	78±2*	47±6
Perinuclear GM130	48±5	51±9	46±12	47±8
Perinuclear Sec31	47±8	37±15	53±14	37±10

Percentage of total population ± s.d. Significant differences (unpaired Student's *t*-test):

*p<0.05.

We observed three levels of Golgi complex reorganization: (I) completely reorganized, i.e. fragmented into elements positioned along the nuclear envelope (Figure 2A, D); (II) partially reorganized (Figure 2B, E); or (III) not at all reorganized (Figure 2C, F). Nocodazole reduced the size of Golgi elements, in agreement with its known effects, but taxol and DW12 did not. DW12 treatments frequently resulted in pericentrin and Golgi elements at the two poles of the nuclei (Figure 2G), a rare distribution in control cells. This polar distribution was even found in cells in which most of the Golgi complex failed to fragment (Figure 2H). Quantitation (Table 2) shows that Golgi complex redistribution is not as complete as pericentrin redistribution in treated cells. Golgi complex reorganization is thus more affected by each treatment than pericentrin reorganization.

**Table 2:** Quantitation of relationship between pericentrin and Golgi complex redistribution, and between Golgi complex and ERES redistribution in cultures grown and differentiated in the presence of microtubule altering drugs.

	control	taxol	nocodazole	DW12
Cells with perinuclear pericentrin that also had perinuclear GM130 (%)	84±3	58±10*	63±7*	63±8*
Cells with perinuclear GM130 that also had perinuclear Sec31 (%)	98±1	86±9	94±1	93±1

Percentage of total population ± s.d. Significant differences (unpaired Student's *t*-test):

*p<0.05.

We previously proposed that ERES determine Golgi complex localization [24] and we therefore monitored any uncoupling of the two. ERES and Golgi complex redistributions were very closely linked (Figure 2I–N) and this relationship was not significantly affected by microtubule alterations (Table 2). Even abnormally localized Golgi complex was surrounded by ERES (arrows in Figure 2I–K). Only occasional myocytes showed GM130 reorganization with a poor or non-existent Sec31 reorganization. Therefore it appears that Golgi elements do not need microtubules to be associated with and localized next to ERES. Although we routinely used 200 ng/ml of nocodazole, we also tested concentrations up to 2000 ng/ml and pre-treatment with cold, with similar results (Figure S4).

Thus we find that the redistribution of centrosome, Golgi complex, and ERES only takes place in myogenin-positive cells and that pericentrin reorganization is upstream of the other components. The redistributions can take place in the absence of dynamic microtubules indicating that these are not essential. However, microtubule manipulations reduce the occurrence of fully normal reorganizations, and this affects the Golgi complex and the ERES more than pericentrin.

### Trafficking of cargo is intact in cells with mislocalized Golgi complex

**Figure 3:**
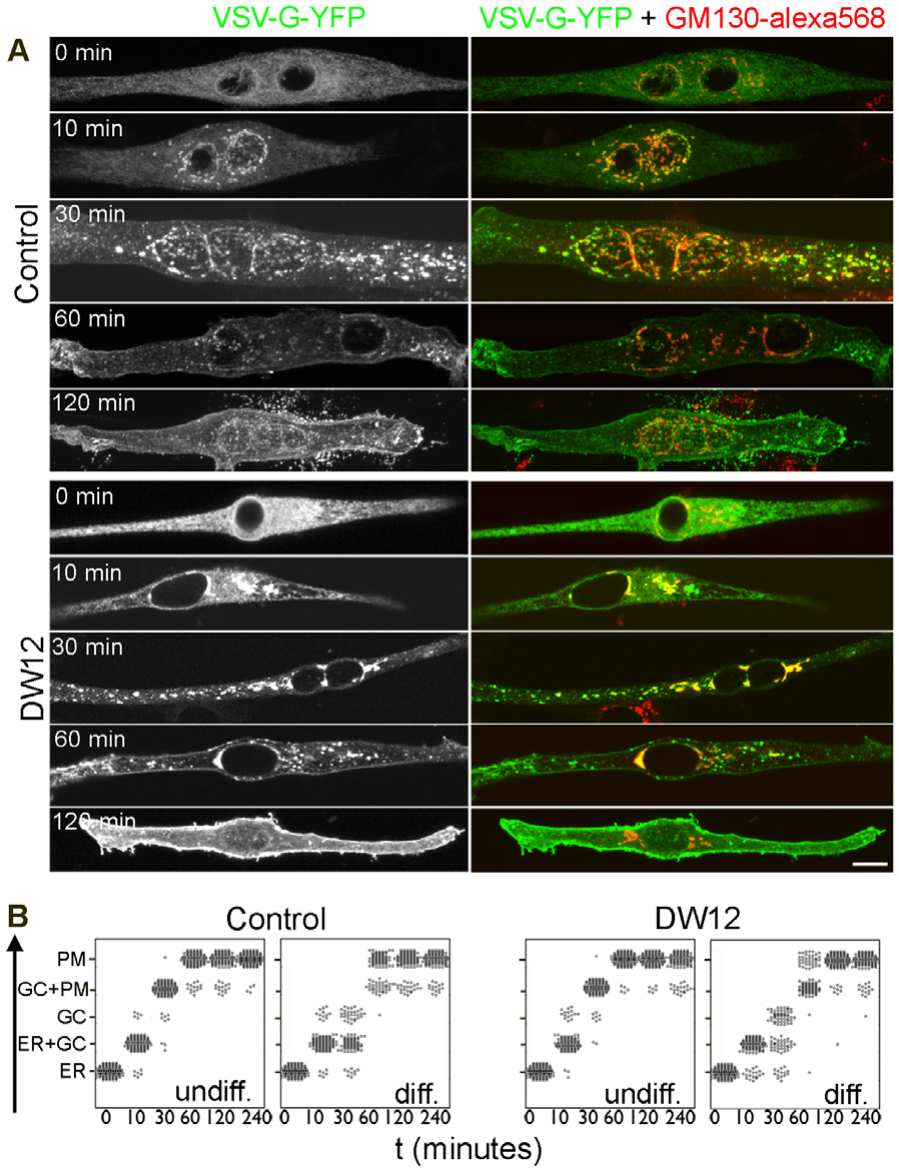
Mislocalized Golgi complex supports normal cargo trafficking. Control and DW12-treated cells were transfected with cDNA for VSV-G-ts_045_-YFP, held at 40°C to keep VSV-G in the ER and switched to 32°C to allow cargo trafficking to the plasma membrane through the Golgi complex. (A) Differentiated cells fixed at the indicated time after switching to 32°C were stained with anti-GM130 (red) while VSV-G is shown in green. Each image is the projection of a confocal z-stack; DW12 treatment does not affect the timing or pattern of cargo transport. Bar: 20 µm. (B) Quantitation of cargo locations. For each time point, the location of VSV-G-ts_045_-YFP was determined in 100 cells (represented by dots). In differentiated cells, DW12 delays cargo clearance from the Golgi complex slightly, but cargo eventually reaches the plasma membrane in control and DW12 cells alike.

As the drug treatments affected both the structure and positioning of Golgi complex and the microtubules normally needed for Golgi complex function, we next assessed their functional consequences, i.e. cargo traffic (Figure 3). We concentrated on DW12-treated cells, as these had both recognizable Golgi complexes and microtubule networks (requirements for secretory traffic), even if altered. We used the model cargo VSV-G_ts045_-YFP, a tagged version of the temperature-sensitive mutant of the vesicular stomatitis virus G protein. Its trafficking kinetics have been documented in several mammalian cell types, including muscle [42], [43], [44], [45]. VSV-G accumulated in the ER at 40°C and was released in both control cells and DW12-treated cells after shifting to 32°C. It reached the Golgi complex after 10 minutes at 32°C and the plasma membrane after 30 minutes. After one hour, the majority of the VSV-G was on the plasma membrane in control and DW12-treated cultures (Figure 3A). The timing of trafficking was very similar to published reports for COS-7 cells [46], [47]. Quantitation showed remarkably little difference between controls and DW12-treated undifferentiated cells, indicating that mislocalized Golgi complexes fully participate in VSV-G traffic (Figure 3B). The only observed effect of DW12 treatment was a slight delay in clearing VSV-G from the Golgi complex in differentiated cells. These results indicate that even mislocalized Golgi complexes linked to abnormally stabilized microtubules can support normal cargo trafficking.

### Developmental stage, not treatment length. predicts success of microtubule recovery after nocodazole treatment

**Figure 4:**
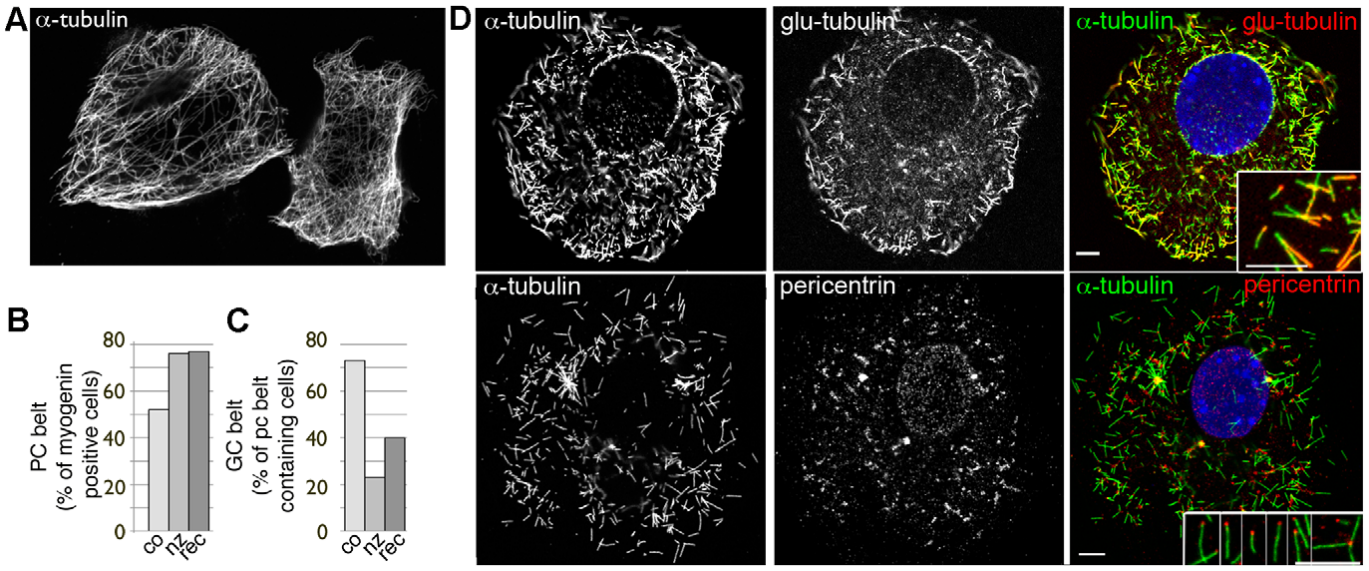
Fast but abnormal recovery of microtubules in cells kept in nocodazole 72 hours through differentiation. After three minutes washout from chronic nocodazole treatment (A), there are abundant microtubules but their pattern is different from that in either myoblasts or myocytes. Quantitation of the redistribution of pericentrin (B) and Golgi complex (C) after recovery (co: control; nz: no recovery; rec: 6 hours recovery). Pericentrin redistribution is unchanged, while Golgi complex organization is improved but does not reach the level observed in control cells. Without washout (D), there are residual short microtubules, many of which contain Glu-tubulin (upper row, with inset). Pericentrin (lower row) is found around the nuclear envelope and in the cytoplasm. The selected cell, which has distinct, albeit very little relocation of pericentrin, also has a cytoplasmic pericentrin pool that consists of a few strongly stained dots at the center of microtubule asters and of many small dots that decorate one end of the short microtubules (inset). Nuclei are counterstained with Hoechst (blue). Main panels: confocal z-stack projections; inserts: individual optical sections. Bars: 5 µm.

**Figure 5:**
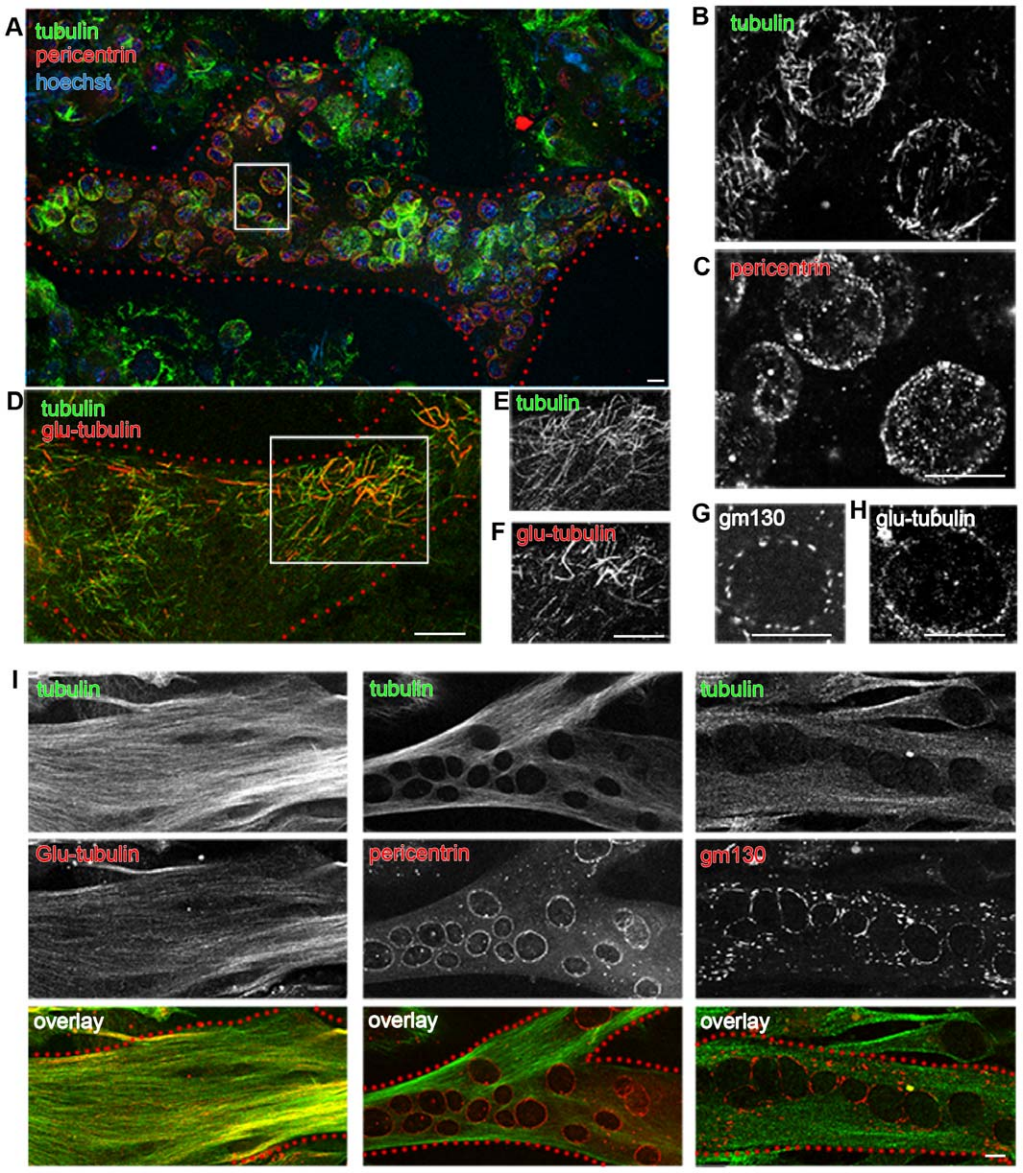
Full recovery of microtubules in cells kept in nocodazole 72 hours after differentiation. Cells were differentiated into myotubes and only then treated with 200 ng/ml nocodazole for 72 hours. At the end of the treatment (A–H), myotubes (outlined in red dotted lines) have lost their elongated shape (A, D) and only contain short microtubules located along the nuclear membranes (B), and in the cell periphery (E). Many of the microtubules contain Glu-tubulin (F). Glu-tubulin is also found in dots along the nuclear envelope (H). Pericentrin (C) and Golgi elements (G) remain localized around the myotube nuclei. Panels (B) and (C) show details from the boxed area in (A); (E) and (F) show details from the boxed area in (D). After 24 hours washout from nocodazole, myotubes are elongated again and have re-established a normal microtubule network (I). Staining patterns for Glu-tubulin (left), pericentrin (middle) or GM130 (right) are undistinguishable from those in untreated myotubes. Images are single confocal optical sections. Bars: 10 µm.

It is conceivable that the Golgi complex reorganization would normalize if nocodazole were washed out after differentiation, assuming that microtubules then would regain the distribution normally found in differentiated muscle cells. We thus removed nocodazole after the usual three-day treatment, and assessed recovery of microtubules (Figure 4). Microtubule regrowth was fast: a complex network was already visible after three minutes (Figure 4A). The presence of these microtubules however did not improve reorganization of the pericentrin or the Golgi complex. The fraction of cells with perinuclear pericentrin was not modified and Golgi complex reorganization was only partially improved (Figure 4B, C). However, the reformed microtubule network differed from both myoblast and myotube normal networks. It contained numerous nodes as if it resulted from nucleation on dispersed sites. Indeed, in cells fixed just before nocodazole was washed out, we observed short residual microtubules, each connected to a pericentrin dot, and each containing Glu-tubulin (Figures S2 and 4D). It looks therefore as if nocodazole washout lets microtubules rapidly reform by elongation from these short residual microtubules. The dispersal of pericentrin to these many short microtubules was found in all nocodazole-treated myoblasts (myogenin-positive or not), but not in untreated cells or in cells treated with taxol or DW12. After 6 hours of recovery from nocodazole, the microtubule network did not show further changes. To assess whether the abnormal recovery is simply due to the length of the treatment with nocodazole (72 hours), we subjected normally differentiated myotubes to the same 72-hour nocodazole treatment. At the end of this treatment, the myotubes had lost their elongated shape (Figure 5A, D). But when nocodazole was washed out, recovery of the cell shape and of a normal-looking microtubule network was complete in 24 hours (Figure 5I). Nocodazole also caused all but small pieces of microtubules to disappear (Figure 5E, F). But unlike in cells differentiating in nocodazole, many short microtubules and some Glu-tubulin staining were concentrated around the nuclei (Figure 5B, H). Perhaps more importantly, pericentrin (Figure 5C) and Golgi elements (Figure 5G) remained positioned around the myotube nuclei. Thus, when differentiation occurs in nocodazole, some irreversible changes affect microtubules. These changes are likely responsible for the incomplete or imperfect redistribution of centrosomal and Golgi complex proteins.

### During normal differentiation, pericentrin relocates gradually and provides a platform for microtubule nucleation, Golgi complex and ERES

**Figure 6:**
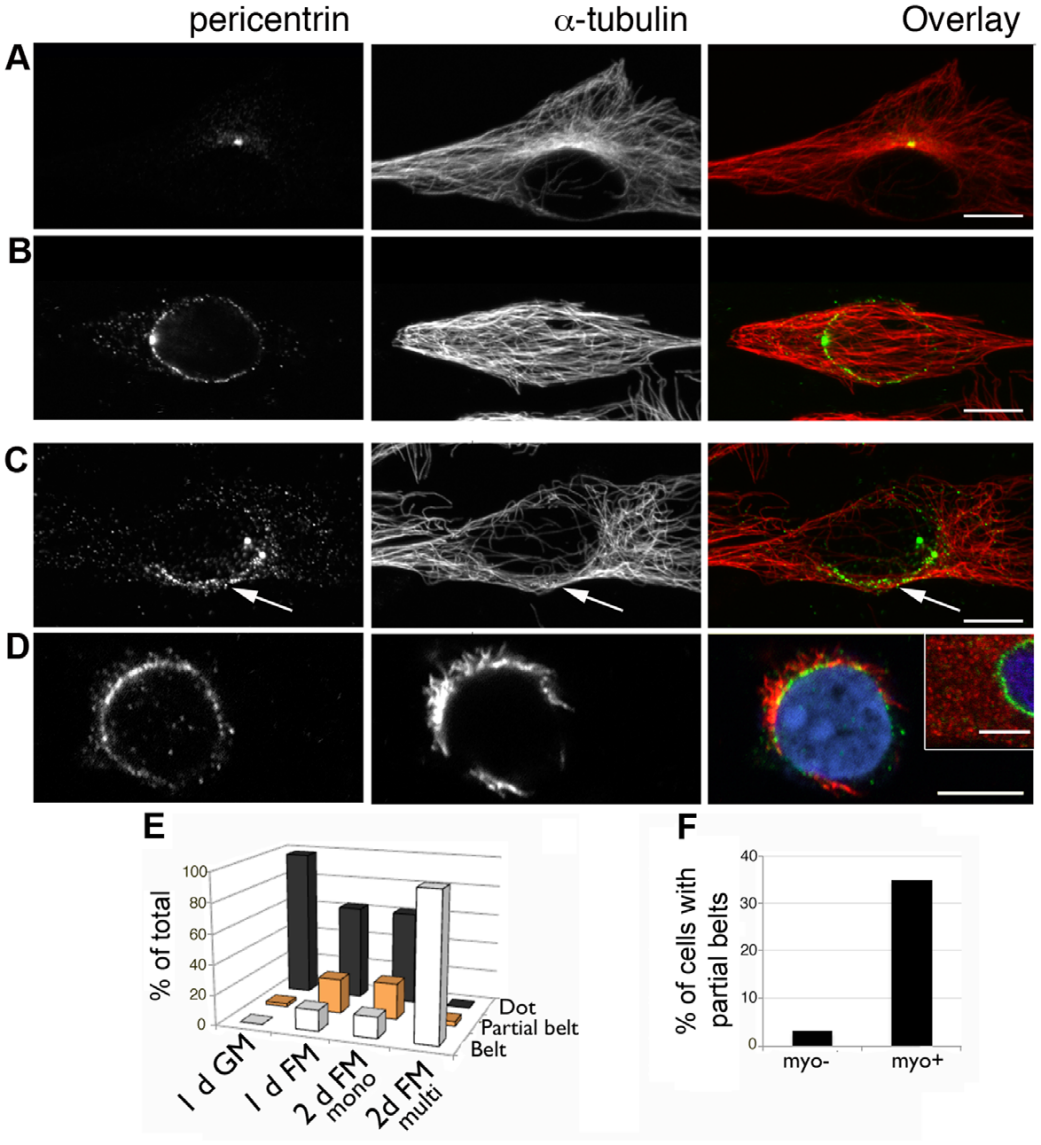
Pericentrin reorganization to the nuclear envelope is gradual. After one day in FM, C2 cultures contain: (A) undifferentiated cells; (B) myocytes with a small pericentrin dot attached to a perinuclear pericentrin belt and longitudinal microtubules; (C) myocytes with a partial pericentrin belt (arrows) and partially remodeled microtubule network. (D) In such cells, microtubule regrowth after complete depolymerization (inset) originates from the partial pericentrin belt. Images are single confocal sections for pericentrin (green) and z-stack projections for tubulin (red). Nuclei are counterstained with Hoechst (blue). Bars: 10 µm; inset: 5 µm. (E) Quantitation of pericentrin morphologies in differentiating cultures and (F) correlation between myogenin expression and pericentrin distribution.

Some rapid cellular events that depend on microtubules, i.e. mitosis, involve a breakdown of existing structures (the normal microtubule network) and *de novo* buildup of another one (the mitotic spindle). The events are regulated by a cascade of signaling events but are not regulated at the transcriptional level. Myogenesis, in contrast, is largely regulated at the transcription level. However, the specific events we are interested in appear gradual. To find out which scenario is involved in organelle redistribution, we searched for cells with partial reorganization of the organelles in untreated cultures (Figure 6A, B). We did find, early in differentiation, elongated cells with a partial nuclear cap of pericentrin emanating from the centrosome (Figure 6C). These cells represent a constant fraction of the myogenin-positive population (Figure 6E, F), as expected from cells entering and leaving a transitional state at similar rates in unsynchronized cultures. In contrast, a steady increase in the number of cells with the partial phenotype would indicate failed progression of differentiation. Even partial nuclear pericentrin caps supported microtubule nucleation (Figure 6D).

**Figure 7:**
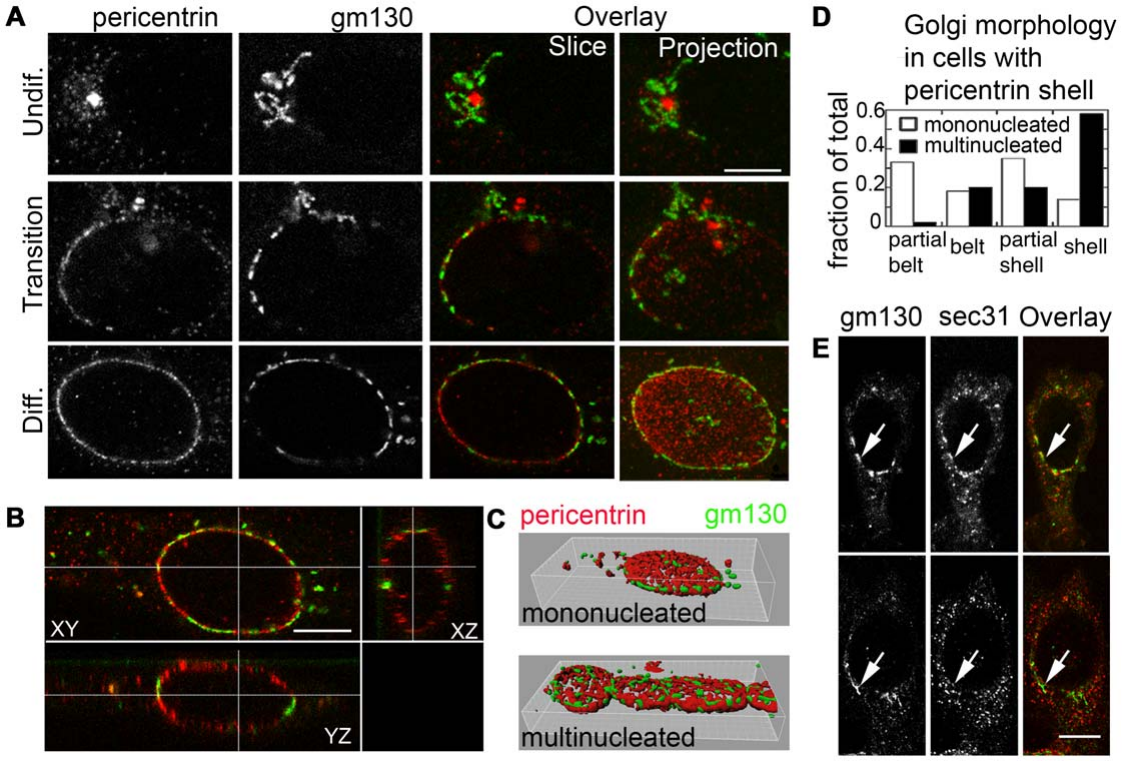
Pericentrin and GM130 reorganize coordinately but occupy distinct domains. (A) During the transition to the differentiated phenotype, GM130 and pericentrin show a similar degree of partial reorganization. (B) Orthogonal views of a myocyte show a full pericentrin shell around the nucleus while GM130 is limited to the equatorial plane. (C) 3D rendering of pericentrin and GM130 in a myocyte (top) and a myotube (bottom). (D) Quantitation of Golgi complex phenotypes in cells with pericentrin shells. (E) ERES clearly clustered at the nuclear envelope (top row), are colocalized with Golgi fragments, but Golgi fragments are found equally with or without ERES clusters (bottom row). The images are confocal optical sections, except for the last row in A (projections of confocal z-stacks). Bars: 5 µm.

Golgi complex reorganization appeared progressive as well (Figure 7A). However, pericentrin expanded more rapidly to a larger domain than the Golgi complex. Furthermore, 3D reconstruction revealed that pericentrin does not form a belt as usually referred to, but a complete shell around nuclei (Figure 7A, C). GM130 was found in different patterns, from partial belts (Figure 7B) to full shells (Figure 7C). The latter became predominant only in multinucleated cells (see quantitation, Figure 7D).

To determine if ERES guide Golgi complex reorganization, as postulated in our model [24], we searched for mismatch between ERES and Golgi complex in transitioning cells. A partial GM130 belt was found as frequently with as without perinuclear ERES clusters (Figure 7E) but perinuclear ERES clusters without a perinuclear Golgi complex belt were not observed.

**Figure 8:**
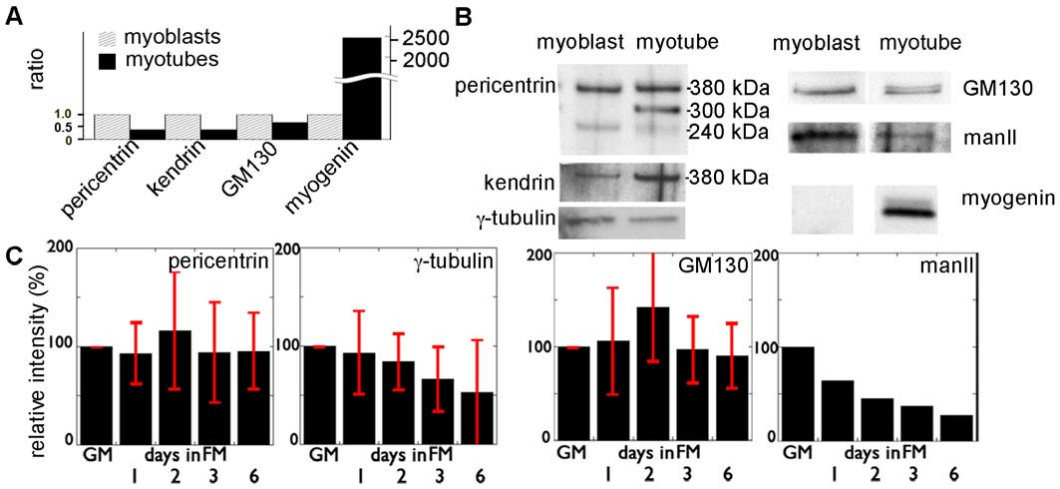
Centrosomal protein and Golgi complex reorganizations do not involve global mRNA or protein level changes. (A) RT-qPCR shows decreases in mRNAs for pericentrin, kendrin, and GM130 during differentiation which are very small compared to the increase in myogenin mRNA. (B) Western blot analysis with anti-pericentrin produces two bands in myoblast and three in myotube homogenates, whereas anti-kendrin (or pericentrin B) recognizes one band, stronger in myotubes. In the pericentrin blot we thus identify the 240 kDa band as pericentrin A, and the 380 kDa band as kendrin. We have not identified the 300 kDa band. Individual blots again GM130 show small changes when compared to the increase in myogenin, while α-mannosidase II (manII), which is absent from adult muscle, shows an expected decrease. (C) Immunoblot quantitation: values from three independent experiments were averaged, normalized to those for cells in GM (± s.d) except for mannosidase (done once).

Quantitative PCR (Figure 8A) demonstrated that centrosome and Golgi complex mRNA changes are two to three orders of magnitude smaller than changes in myogenin mRNA. In addition, we found no differentiation-related shift between the isoforms of pericentrin (A and B or kendrin [48]). At the protein level as well, changes in centrosomal and Golgi complex proteins are modest compared to those for myogenin (Figure 8B, C). We conclude that myogenic reorganization of the centrosome and Golgi complex involves a gradual transformation and relocation of the organelles.

### Microtubules constantly nucleate from myotube nuclei

We have proposed a model [24], which explains the presence of the Golgi complex around the myotube nuclei by the coalescence of the microtubule nucleation sites and of the ERES. Newly formed pre-Golgi complex vesicles that emerge from the ERES and normally move retrogradely to microtubule minus ends, stay put since they are already at the microtubule minus ends. But this model hinges on microtubules constantly nucleating from the nuclear envelope area [24], [49] and this has not been shown in unperturbed myotubes. We and others have previously identified the nuclear membrane area of myotubes as a bona fide microtubule nucleation site only during recovery from complete depolymerization [49], [50]. The high density of microtubules in unperturbed cells hampers tracing their origin.

**Figure 9:**
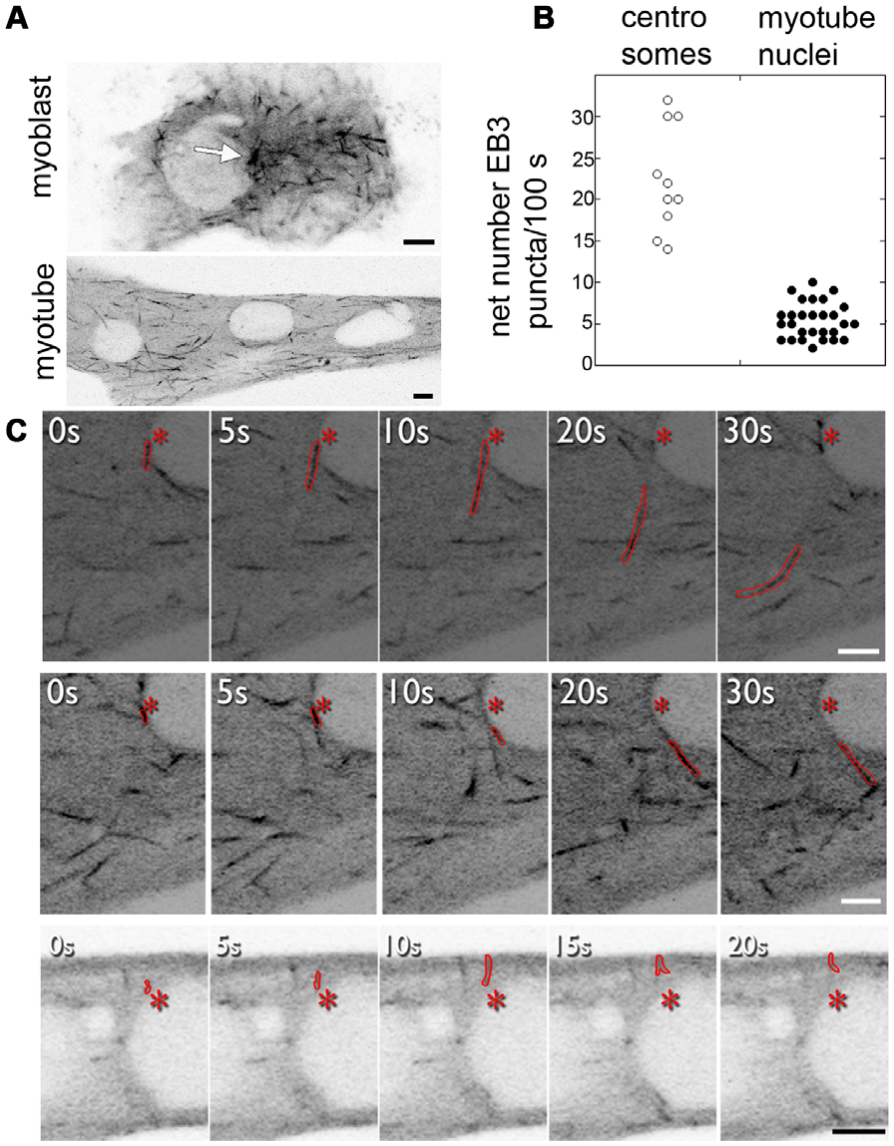
EB3-GFP tracking reveals microtubule nucleation from the nuclear membrane of myotubes at steady-state. (A) Single frames from time-lapse recordings of EB3-GFP in a myoblast (Movie S1) or in a myotube (Movie S2). The arrow in (A) points to the centrosome. (B) Quantitation: we measured 22.4±6.4 nucleations per 100 seconds for centrosomes (n = 10), and 5.4±2.2 per 100 seconds per nucleus for myotubes (n = 28). (C) Details from a myotube recording showing EB3 puncta (outlined in red) moving away from a nucleus (asterisk marks the initial location of the punctum). Bars: 5 µm.

**Figure 10:**
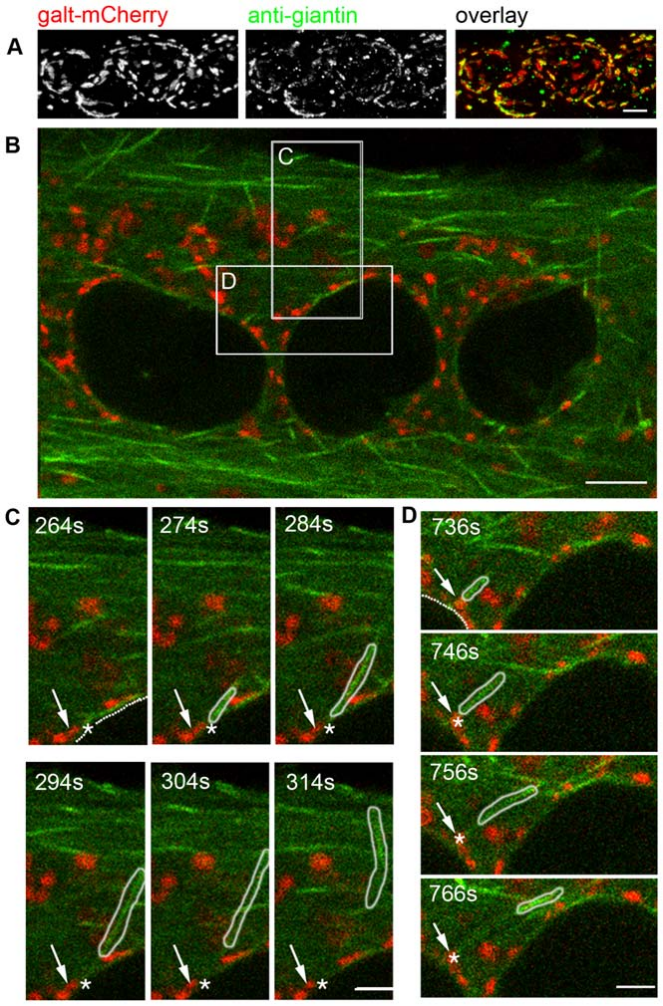
No obligatory relationship between microtubule nucleation and Golgi complex fragments along the myotube nuclear membrane. (A) GalT-mCherry colocalizes with the Golgi marker giantin (projection of a confocal z-stack). (B) Single frame from time-lapse recordings of EB3-GFP (green) and GalT-mCherry (red) in a doubly transfected myotube (Movie S3). (C, D) EB3 puncta (outlined in white) moving away from a nucleus (delineated by dotted line). The starting point of the punctum (star) in (C) is near but distinct from a Golgi element (arrow) while in (D) the punctum starts from a Golgi element. Boxes in (A) indicate areas enlarged in C, D. Bars: 5 µm.

We now use a GFP chimera of the microtubule plus-end protein EB3 to observe microtubule dynamics in live muscle cells (Figure 9). In myoblasts, EB3-GFP puncta move outward in the well-known fountain-like pattern, at the maximum frequency of 32 puncta per 100 seconds per centrosome (range: 14–32 per 100 seconds; Figure 9A,B, Movie S1). In myotubes, in contrast (Figure 9A, B, Movie S2), EB3-GFP puncta have no concerted movement, in agreement with the published behavior of EB1 [51]. Rather, EB3-GFP puncta move toward the plasma membrane and toward the myotube ends, often crossing paths. We were able to track EB3 puncta originating from myotube nuclei (Figure 9C). The maximum number of puncta observed per 100 seconds per nucleus was 10 (range: 1–10 per 100 seconds, Figure 9B). Thus there is steady-state microtubule nucleation at the myotube nuclear envelope. Trajectory analysis showed that some cytosolic spots repeatedly produce EB3 puncta. These spots probably represent centrosomal remnants, nucleation-competent cytoplasmic elements containing a centriole and centrosomal proteins [49]. To determine whether steady-state nucleation at the nuclear membrane is associated with Golgi elements, EB3-GFP and the Golgi marker GalT-mCherry were co-transfected. GalT-mCherry faithfully targets the Golgi complex in myotubes, as verified by anti-giantin staining (Figure 10A). If Golgi elements nucleate microtubules, EB3-GFP trajectories should start from GalT-mCherry-labeled Golgi stacks. As shown in Figure 10B–D and Movie S3, this was not the case. Microtubule nucleation was observed from areas not labeled with Galt-mCherry.

**Figure 11:**
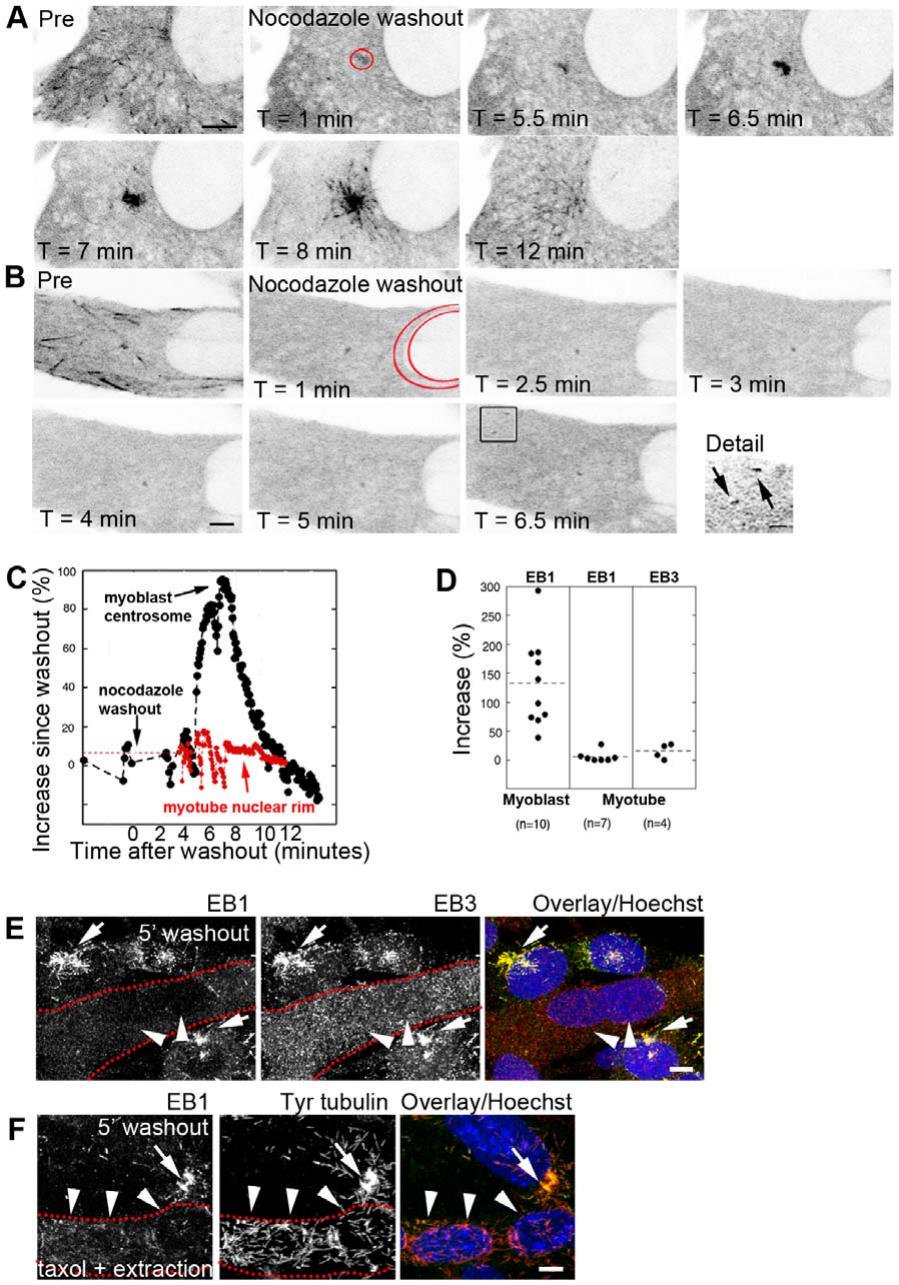
Timelapse recordings of EB1- or EB3-GFP highlight differences between myoblasts and myotubes after washout from acute nocodazole treatment. Myoblasts and myotubes expressing EB1-GFP were imaged before, during, and after treatment with nocodazole (1 hour, 5 µg/ml). (A) In myoblasts, EB1-GFP comets are present before treatment (A, Pre), absent during nocodazole treatment (not shown) and at the start of washout (A, t = 1 min). Over the next several minutes, EB1-GFP accumulates in the centrosomal region (A, t = 5.5–8 min). When the return of EB1-GFP comets throughout the cytosol indicates the restart of steady-state microtubule growth, the level of EB1-GFP at the centrosome returns to its initial value (A, t = 13 min). (B) There is no such increase in the EB1-GFP concentration at myotube nuclear rims, despite the return of EB1-GFP comets in the cytosol at 6.5 minutes (see detail of boxed area), indicating that microtubule growth has commenced. (C) Analysis of the level of EB1-GFP in the areas outlined in red in the images shown in A (black) and B (red). (D) Quantitation of EB1-GFP and EB3-GFP levels after nocodazole washout in myoblast centrosomes and myotube nuclear rims. (E) To exclude that the results in B are due to problems with the GFP-tagged constructs, the same treatment was carried out on untransfected cultures. Five minutes after nocodazole washout, cells were fixed and stained for EB1 and EB3. Again, neither accumulates at myotube nuclear rims (arrowheads), while both are prominent at myoblast centrosomes (arrows). (F) When a microtubule stabilization protocol combining detergent extraction and taxol is used before fixation, only very weak EB1 staining is found near myotube nuclear rims (arrowheads), despite the presence of many short microtubules in this location. Bright staining is present at centrosomal regions (arrows) and on small microtubules in myoblasts' cytoplasm. (E, F: dashed lines outline myotubes). Bars = 5 µM (main panels), 1 µM (detail).

The number of EB3 puncta originating from myotube nuclei in unperturbed cells appeared modest when compared to the density of small microtubules observed after recovery from complete microtubule depolymerization [49], [50]. To understand this difference, we imaged EB3-GFP expressing cells on a confocal microscope at 37°C throughout nocodazole treatment and recovery. Unlike myoblast centrosomes which produce a burst of EB comets immediately following washout (Figure 11A, Movies S4 and S5), myotube nuclear membranes, in the same experimental conditions, do not show comets emerging from the nuclear rim (Figure 11B, Movies S6 and S7). Moreover, comets could only be found after several minutes of recovery,and in the cell periphery. Quantitation of EB fluorescence showed that the myoblast centrosomal area becomes transiently brighter after washout while myotube nuclear areas do not (Figure 11C, D). To rule out that the lack of EB comets in myotubes is due to the GFP tags on the EB proteins, we examined untransfected cells fixed 5 minutes after nocodazole washout and stained with anti-EB1 or -EB3. We found abundant EB1 or EB3 around the centrosomes of myoblasts, but practically none at myotube nuclear rims (Figure 11 E). Since EB1 is normally in constant exchange between microtubules and cytosol, we wanted to rule out that this exchange, possibly accelerated in myotubes, explains the absence of EB staining during microtubule recovery. A pre-fixation protocol combining a brief detergent extraction with taxol to stabilize EB proteins and preserve the accurate state of the *in vivo* microtubule assembly (see Methods and [52]) allowed us to visualize small newly nucleating microtubules and EB1 simultaneously, 5 minutes after nocodazole washout (Figure 11F). Both myotubes and myoblasts show many small microtubules (Figure 11F middle panel). A weak signal along the nuclear membrane of the myotubes indicates the presence of a small amount of EB1, but the staining remains much weaker than in myoblasts.

Thus we have demonstrated that nucleation of microtubules from the nuclear membrane of myotubes is constant, and we have uncovered an unexpected lack of EB proteins on small microtubules emerging at the earliest times after removal of nocodazole.

## Discussion

It has been difficult, so far, to detect a hierarchy in the different reorganizations that take place during myogenesis because they occur seemingly simultaneously. Here we have attempted to uncouple them by challenging the cells to differentiate in the presence of drugs that affect microtubules. We find that each of the subcellular systems we examine, i.e. centrosome (MTOC), Golgi complex, and ERES, is able to relocate despite microtubule alterations. We also find that the relocation of the centrosomal proteins paves the way for the relocation of the other systems. Furthermore, centrosomal proteins reorganize more completely than Golgi complex and ERES sites in the absence of a dynamic microtubule network. We conclude that direct transport along dynamic microtubules is not involved in the transfer of centrosomal proteins to the nuclear envelope, but that it plays some role in Golgi complex and ERES reorganization.

Some of the cells that differentiate in microtubule-affecting drugs reach a state of partial organelle redistribution. We could not improve the outcome by washing the drug away. This experiment concentrated on nocodazole treatment, because its effects are known to be reversible within a matter of minutes to hours. Less is known of reversal of the effects of taxol and DW12. Our observations highlight irreversible changes that affect the reciprocal relations between organelles and microtubules during myogenesis.

Our findings support our previously published model [24] in which Golgi complex fragmentation and reorganization during myogenesis involve microtubule-dependent retrograde trafficking through the ER, whereas centrosomal proteins move from centrosome to nuclear membrane through a more direct, microtubule-independent path. Our model also predicted that ERES relocate ahead of and guide the Golgi complex. We have not been able to prove this because microtubule manipulations did not uncouple them sufficiently. The observation of a few cases when one reorganized without the other suggests that ERES redistribute independently of the Golgi complex, possibly consolidating around ER areas rich in Golgi complex enzymes, in agreement with Guo and Linstedt [53]. ERES fusion and fission have been studied extensively [54] but ERES positioning is poorly understood because no other cell types that we know of show specific ERES localization in physiological conditions.

Redistribution of centrosomal proteins is enhanced rather than impeded by treatment with nocodazole and taxol. Since dynein is necessary to bring pericentrin and γ-tubulin to centrosomes [28], [29], [55], nocodazole may free the two proteins from the MTOC, possibly explaining how pericentrin dots come to decorate one end of the short microtubules that remain in the cells after chronic nocodazole treatment (Figure 4A). Taxol similarly enhances redistribution of centrosomal proteins but DW12 treatment, which inhibits GSK3-β, does not. Both taxol and DW12 stabilize microtubules, but the extent and pattern of stabilization differ. In addition, GSK3-β has been indirectly linked to microtubule anchoring at the centrosome through its control of Bicaudal-D, altering the binding of Bicaudal-D to ninein and ninein transport to the centrosome [56].

In the present work, we have also clarified key aspects of the organelle reorganization in normal cells. We show that MTOC reorganization is dependent on myogenin expression and is upstream of and essential for Golgi complex reorganization. We also show that reorganization is progressive, and that even small patches of centrosomal proteins along the nuclear membrane can support microtubule growth and attract the Golgi complex (Figure 5 and [50]). Pericentrin therefore determines or at least identifies a domain of the nuclear membrane competent for microtubule nucleation, Golgi complex and ERES localization. This correlation persists through muscle maturation since Golgi elements in muscle fibers are associated with concentrations of pericentrin [25], [26] at the nuclear poles and in the cytoplasm (Oddoux et al., in preparation). We also noticed that pericentrin forms a full perinuclear shell in myocytes. This possibly occurs by self-assembly as suggested by Srsen et al. [43], and would explain pericentrin's independence from normal microtubule organization.

Quantitative PCR and immunoblotting experiments indicate that relocation of the organelles during myogenesis does not involve transcriptional or translational activation of the genes involved and must therefore be regulated post-translationally. For pericentrin at least we expected significant changes at the protein level. Immunofluorescence gives the impression of a vastly expanded staining when comparing the small centrosome to the full perinuclear shell, but changes during differentiation in the amount of pericentrin and its message are in fact small. We ruled out that differentiation involves a switch from pericentrin to kendrin (pericentrin A and B, respectively) and did not detect pericentrin S, an isoform predicted from RNA analysis to be abundant in muscle [48], [57] but never actually reported.

We also revisited microtubule nucleation. Previous studies, including ours [1], [49], [50], have investigated this process only during recovery from microtubule depolymerization. For most cells this is justified, since the microtubule pattern during recovery is similar to that at steady-state, but in myotubes this is not the case. We show, for the first time, that microtubules constantly originate from the nuclear membrane of myotubes at steady-state. This is important because it explains the Golgi complex localization around the nucleus. Nucleation from myotube nuclei is less frequent than from centrosomes, even after taking into account the much larger nuclear surface. Furthermore, we have uncovered a basic difference between myotubes and myoblasts (and, consequently, other proliferating cells) during recovery from microtubule depolymerization. The short microtubules reforming around myotube nuclei appear initially devoid of the plus-tip proteins EB1 and EB3, unlike microtubules reforming from centrosomes. Centrosomes contain EB proteins [58] whereas nuclear membranes have not been shown to accumulate them. In myotubes, EB proteins must therefore be recruited from the cytoplasmic pool to the newly forming microtubules along the nuclear membrane. In myoblasts, EB proteins are immediately available to the forming microtubules. EB proteins are considered core components that link other plus-tip proteins to microtubules (see [59]). We are not aware of another situation in which newly forming microtubule plus-tips contain neither EB1 nor EB3. We conclude that there are structural and functional differences between centrosomes and unconventional MTOC, a difference relevant to several non-muscle cell types as well.

Time-lapse recordings of EB3-GFP in myotubes give at first an impression of random movement, but there are also repetitive trajectories and crossing points (Movie S2), suggesting that EB3-GFP labels individual microtubules growing along stationary bundles. Golgi complex elements were occasional but not sole sources of microtubule nucleation at the nuclear envelope, indicating that Golgi elements are positioned at the nuclear membrane because of microtubule nucleation rather than the reverse.

In addition to the traditional nucleation and anchoring observed in myoblasts, other processes may take place in myotubes such as anchoring after nucleation at another site [33] and regrowth of microtubules from existing stable tubulin seeds [60]. Regrowth from existing seeds seems the most likely explanation for the short Glu-tubulin-containing microtubules depicted in Figure 4D.

Uncoupling subcellular changes taking place during muscle differentiation has allowed us to establish centrosome/MTOC reorganization as the first step of the systems that we monitored and to show that the changes are remarkably resistant to microtubule disruptions. Finding out how pericentrin and other MTOC components in muscle are linked to nuclei is one of the most interesting questions ahead. It seems likely that transmembrane proteins of the nuclear membrane, such as the nesprins (reviewed in [61]), which mediate interactions between centrosome and nucleus, must be involved.

Changes in Golgi complex morphology or placement are automatically assumed to affect function negatively. Here we show, however, that mislocalization caused by treatment with a GSK3-β inhibitor barely slows down cargo trafficking, an essential Golgi complex function. Rahkila et al. [43] reported that VSV-G is retained in an intracellular compartment after muscle cell differentiation. We did not observe such retention, perhaps because of differences in the experimental conditions: Rahkila et al. [43] used full virus infection to express VSV-G in L6 cells, whereas we used cDNA transfection to express VSV-G in C2 myoblasts and myotubes.

Microtubules play structural roles in muscle-specific events such as myoblast elongation, fusion or sarcomere formation [62], [63], [64] and they are essential to differentiation [65], [66]. They also play a role in processes that are not muscle-specific but are important in muscle, such as macroautophagy [22], [23]. The fact that we find only minor defects in cargo trafficking when microtubule organization is altered and the Golgi complex is mislocalized, suggests that cells can tolerate some level of disturbance of microtubules and related organelles. This would be good news for conditions such as Pompe disease, or Duchenne Muscular Dystrophy, in which Golgi complex and microtubules are affected [18], [19].

## Materials and Methods

### Antibodies and Reagents

Mouse anti-GM130 antibodies were from Transduction Labs (Lexington, KY), anti-giantin from Covance (Denver, PA), anti-myogenin from Dako (Carpinteria, CA), anti-GAPDH from Abcam (Cambridge, MA), anti-α-tubulin and anti-γ-tubulin from Sigma (St. Louis, MO) and rabbit anti-detyrosinated tubulin from Millipore (Billerica, MA). Rabbit anti-pericentrin and anti-giantin were from Covance, and anti-GAPDH from Novus (Littleton, CO). The following antibodies were generous gifts: rabbit anti-α-mannosidase II (Dr. K. Moremen, University of Georgia, Athens, GA), rabbit anti-Glu-tubulin (Dr. G. Cooper IV, Veterans Administration Medical Center, Charleston, SC), chicken-anti-kendrin (Dr. T. Davis, University of Washington, Seattle, WA), and rabbit anti-Sec31/p137 (Dr. F. Gorelick, Yale University School of Medicine, New Haven, CT). Alexa488- and Alexa568-conjugated secondary antibodies were purchased from Invitrogen (Carlsbad, CA). HRP-conjugated anti-mouse and anti-rabbit IgG were from Bio-Rad (Hercules, CA) and HRP-conjugated anti-chicken IgY, together with BlockHenII, were from Aves Labs, Inc. (Tigard, OR). Hoechst 33342, nocodazole and taxol were from Sigma. DW12 was a kind gift from Dr. E. Meggers (University of Pennsylvania, Philadelphia, PA).

### Cell culture and drug treatments

Mouse C2 skeletal muscle cells were cultured in growth medium (GM) and differentiated in fusion medium (FM) as described [24]. Drugs were added 4 hours after plating to achieve 200 ng/ml (0.66 µM) nocodazole or 50 nM taxol. In some experiments cells were placed on ice for 30 minutes after the addition of nocodazole and then warmed up to at 37°C in the drug. Because nocodazole and taxol inhibit cell proliferation, dishes for these treatments received twice the control cell number. For nocodazole washout, cells were fixed or rinsed three times in PBS, and further cultured in drug-free media at 37°C for the times indicated before fixation. The drug DW12 was used at 100 nM. This organo-ruthenium compound specifically inhibits GSK3-β and, like the broad inhibitor lithium chloride, leads to local microtubule stabilization [37], [67], [68].

The Mitotic Index [MI = (cells in prophase+anaphase+metaphase+telophase)/total cell count] of the C2 culture, calculated on 3 separate samples of 400 cells, was 0.042±0.006 (mean ± standard deviation, s.d.).

### Immunofluorescence and microscopy

Cells were fixed in 100% methanol at −20°C for 6 minutes, blocked in PBS containing 1% bovine serum albumin, 2% horse serum and 3% normal goat serum for one hour, incubated with primary antibodies for 1–2 hours, and with secondary antibodies for 45 minutes (all at room temperature), counterstained with Hoechst 33342, and mounted in Vectashield (Vector Laboratories, Burlingame, CA) or in home-made mounting media consisting of p-phenylenediamine in 80% glycerol. Wide-field immunofluorescence images were collected on a Leica DMR, using a 63× 1.32 N.A. oil immersion objective lens and a Hammamatsu C4742-95 camera driven by IVision software (BioVision Technologies, Exton, PA). Confocal images were collected either on a Zeiss 510 LSM using a 63× 1.4 N.A. oil immersion objective lens and multi-track configurations, or on a Leica SP5 confocal microscope with a 63× 1.4 N.A. oil immersion objective lens, using sequential scanning. The figure legends specify whether images are single optical planes or projections of stacks of optical sections throughout the cell (z-stacks). Images were exported in 8-bit TIF format and linearly adjusted using Adobe Photoshop.

### Quantitation of reorganization

To assess the effect of a range of nocodazole concentrations on subcellular organization, cells were grown for 48 hours in GM and differentiated in FM for 24 hours with the indicated concentration of nocodazole. Images were collected every two hours using an IncuCyte image system (on a kind loan from Essen Instruments, Ann Arbor, MI). Cell confluence was plotted using the manufacturer's software. Since C2 cells kept in microtubule-disrupting drugs fuse poorly, we analyzed differentiated but unfused cells (myocytes), even in control cultures. For Tables 1 and 2, we scored 600 myogenin-positive cells per time-point for the presence of perinuclear staining (belt), by observation on a wide-field microscope. Cells with the majority of the marker in a perinuclear belt were classified as reorganized. Cells with the marker divided between perinuclear belts and additional locations were classified as partially reorganized. Cells without perinuclear belts were classified as not reorganized. In the next step, cells with reorganized pericentrin were selected and analyzed for Golgi complex morphology. Finally, cells with reorganized Golgi complex were analyzed for reorganization of ERES. Data are expressed as means ± s.d. An unpaired Student's *t*-test was used for statistical analysis (Excel software). For analysis of reorganization after nocodazole washout, 400 cells in two independent experiments were analyzed for the presence of perinuclear pericentrin or GM130. The analysis of pericentrin reorganization in control cultures (Figures 5 and 6) was done in 200 myogenin-positive and 200 myogenin-negative cells at each indicated time point. Additionally, confocal z-stacks were collected of cells with reorganized pericentrin (one day in FM), and in those, the morphology of the Golgi complex was classified using 3D renderings in Imaris software (Bitplane, Saint Paul, MN).

### Microtubule Nucleation Assay

Cells were incubated in 5 µg/ml nocodazole on ice for 30 minutes to sever microtubules and then at 37°C for 30 minutes to depolymerize microtubules completely. To regrow microtubules, cells were quickly rinsed twice in warm PBS and incubated for the indicated times in GM or FM at 37°C. For better visualization of the regrowing microtubules, cells were extracted at room temperature for one minute in PHEM buffer (60 mM piperazine-N,N-bis-2-ethanesulfonic acid, 25 mM HEPES, 10 mM EGTA, 2 mM MgCl_2_, pH 6.9) containing 1% Triton X-100 and rinsed in PHEM buffer for 20 seconds before fixation. For visualization of both EB proteins and small microtubules right after nocodazole washout, cells were incubated for one minute in 4 µM taxol in a buffer containing 0.5% Nonidet P-40, 20 mM Tris HCl, 140 mM NaCl, 1 mM MgCl_2_, 2 mM EGTA (pH 6.8 [52]) at RT before fixation.

### Cell homogenates and Western analysis

Cells growing on Petri dishes were trypsinized, collected by centrifugation, washed twice, and taken up in sample buffer (National Diagnostics, Atlanta, GA). The samples were assayed for protein concentration (Pharmacia 2d Quant kit, Peapack, NJ); proteins were separated on pre-cast tris acetate or bis-tris gradient gels (Invitrogen) at 100 V for one hour or at 200 V for 35 minutes and transferred to nitrocellulose membranes at 51 V for 4 hours or at 20 V overnight at 4°C. The membranes were blocked in 5% milk powder in TBS for one hour (except for 2% FBS and 1% NGS in TBS for α-mannosidase II, or 7.5% BlockHen, 5% NGS in PBS for anti-kendrin), incubated with primary antibodies for two hours and with HRP-conjugated secondary antibodies for one hour at room temperature. The peroxidase activity was revealed with Pierce Supersignal West (Rockford, IL) and captured on Kodak BioMax MR film (Rochester, NY). Films were scanned on a UMAX scanner (Dallas, TX) and band intensities were measured with ImageJ software (written by W.S. Rasband, National Institutes of Health, and available at http://rsb.info.nih.gov/ij/). Molecular weights for bands on pericentrin films were estimated using HiMark Prestained HMW protein standard (Invitrogen). Polynomial curve-fitting of the relative migration distances (Rf) was done in Kaleidagraph software (Synergy Software, Reading, PA).

### Quantitative RT-PCR (RT-qPCR)

Total RNA was purified using Trizol (Invitrogen) and samples (100 ng) were reverse transcribed using High Capacity cDNA Reverse Transcription Kit (Applied Biosystems, Foster City, CA). cDNA samples were diluted 10-fold and used for qPCR using SYBR Green (Applied Biosystems). For quantitation, values were normalized over internal control (GAPDH) and represented relative to myoblast levels. Primers used are, 1) for sequences shared by pericentrin & kendrin: forward 5′ GTCTGTCCAGGAGGAGGGTTC, reverse 5′ AGGTCAGTCACATCCAGTTCAC 3′; 2) for Kendrin specific: forward 5′ AACTCACTCGAGATGACCTTCTG 3′, reverse5′ GGCAAACTTCTCCATGATCTCT 3′; 3) for GM130: forward 5′ GCCAAGAAAAAGCTGAGAGAGT 3′, reverse 5′ GGACACCAGCACCTTCAGAAT 3′; 4) for GAPDH: forward 5′ AACATCAAATGGGGTGAGGCC 3′, reverse 5′ GTTGTCATGGATGACCTTGGC 3′.

### Secretory cargo trafficking assay

Cells were plated on coverglass and DW12 (100 nM) was added after 4 hours. After another 16 hours, the cells were transfected with 1.2 µg of VSV-G_ts045_-YFP cDNA using FuGENE transfection reagent (Roche, Indianapolis, IN) and incubated for 24 hours at 37°C. The cultures were then switched to 40°C. Control cultures grow and fuse normally at 40°C. Some cultures were kept at 40°C for one day, then incubated for various times at 32°C for cargo release, and fixed (undifferentiated cultures). Others were cultured in FM at 40°C for two days, then incubated for various times at 32°C, and fixed (differentiated cultures). The location of pools of VSV-G was analyzed in 100 cells per time-point, using 5 possible categories: ER, Golgi (identified by anti-GM130 staining), plasma membrane, and a combination of these.

### EB3-GFP imaging

Cells in MatTek dishes (MatTek, Ashland, MA) were transfected one day after plating with one µg of EB3-GFP cDNA (a kind gift from Dr. A. Akhmanova, Erasmus Medical Center, Rotterdam, the Netherlands) using FuGENE transfection reagent. Undifferentiated cells were imaged one day post-transfection, differentiated cells after two days in FM. Image series of living cells were collected at 37°C on a Zeiss 510 LSM, using a 63× 1.4 NA objective, at one frame/second. Files were exported in 8-bit TIF format, and images were processed by Kalman filtering in ImageJ. EB3-GFP tracks were analyzed in the nuclear area of two myoblasts (one nucleus each) and three myotubes (with combined total of eight nuclei). In stacks of 100 frames ( = 100 seconds), EB3 puncta arriving at the centrosome (arrow in undifferentiated cell) or at a myotube nucleus were counted as *N_a_* and EB3 puncta leaving from it were counted as *N_l_*. Each dot in the graph represents the net number of puncta (*N_l_*−*N_a_*).

Galactosyltransferase tagged with mCherry (GalT-mCherry) was constructed from GalT-eGFP [14] and p-mCherry N1, a kind gift from Dr. G. Patterson (NIBIB, NIH, Bethesda, MD). Myoblasts were transfected with EB3-GFP and GalT-mCherry plasmids using FuGENE and differentiated into myotubes. Time-lapse recordings of living cells (1 frame/2 seconds) were collected at 37°C using a Leica SP5 confocal microscope.

### Analysis of EB1-GFP or EB3-GFP at centrosomes and nuclear rims after nocodazole washout

Cells in 35 mm MatTek dishes were imaged on a Zeiss 510 Meta microscope at 37°C at one frame/second, then nocodazole was added as a 2× stock (5 µg/ml final concentration) and the imaging was slowed to 1 frame/5 min. After 1 hour, the dish was washed 3 times. Imaging was restarted at 1 frame/second as soon as the original focus level was restored. Image series were exported as TIF files. For illustrations, images were inverted and non-linear adjustments for brightness were made in Adobe Photoshop. To show details of comets after the washout, images for the separate panels were further enhanced in a non-linear manner. Intensity changes in centrosomal regions (circular) and at nuclear rims (free-hand shape outward about 2 µm from the edge) were quantified with ImageJ on unprocessed files. Normalized values (to the first frame after washout) were plotted. The maximal increases measured for myoblast centrosomes and myotube nuclei were plotted using Kaleidagraph. Considerable changes in the position of the myotube nuclei, necessitating refocusing, made the myotube curves less smooth than the myoblast curves.

## Supporting Information

Figure S1 **Effects of microtubule-affecting drugs on C2 cultures.** (A) Optimization test for nocodazole concentration; cultures were imaged every 2 hours. Cell proliferation is arrested from 50 ng/ml on. (B) State of microtubules (α-tubulin, green) and microtubule stabilization (Glu-tubulin, red) before the switch to FM to initiate differentiation. (C) Myogenin expression in control and treated cultures after 2 days in GM, before the switch to FM to initiate differentiation. Wide-field images. Bar: 10 µm.
(TIF)

Figure S2 **Cell shape and state of the microtubules in C2 cells differentiated in microtubule-disrupting drugs.** Control cultures show many elongated cells and multinucleated myotubes with longitudinal microtubules (α-tubulin, green). Microtubule stabilization is reflected by Glu-tubulin staining (red, arrowheads), which increases in differentiated cells. In cultures treated with taxol (50 nM), the cells do not fuse but are large and elongated with high levels of Glu-tubulin. DW12 (100 nM) has no global effect on the microtubule network but, in most cells, increases Glu-tubulin next to the nucleus, and inhibits fusion. Nocodazole (200 ng/ml) prevents both elongation and fusion. Only very short microtubules are left, which partly stain for Glu-tubulin. Images are confocal z-stack projections. Outlined areas in the third column are shown enlarged in the last column. Nuclei are counterstained with Hoechst (blue). Bars: 25 µm.
(TIF)

Figure S3 **Organization of centrosomal proteins, Golgi complex and ERES in control undifferentiated cells.** (A) Golgi complex identified by GM130 staining (top, green) and centrosome identified by pericentrin staining (middle, red). (B) GM130 staining (top, green) and ERES identified by Sec31 staining (middle, red). Bottom row: overlay with nuclear counterstain (blue). Wide-field images. Bar: 10 µm.
(TIF)

Figure S4 **A large range of nocodazole concentrations and cold affect subcellular reorganization similarly.** Cells with or without cold pretreatment were differentiated in FM with nocodazole for one day and myogenin-positive cells were assessed for the presence of perinuclear pericentrin belts. (A) All concentrations increased the fraction of cells with reorganized pericentrin. Cold pre-treatment did not have an additional effect. (B) Examples of cells with reorganized pericentrin, GM130 and Sec31 in cultures treated with cold and 2000 ng/ml nocodazole. Wide-field images. Bar: 10 µm.
(TIF)

Movie S1 **Time-lapse recording of a C2 myoblast expressing EB3-GFP.**
(MOV)

Movie S2 **Time-lapse recording of part of a C2 myotube expressing EB3-GFP.** Puncta originating at the nuclear envelope indicate steady-state growth of new microtubules from this area. There is also constant longitudinal movement of puncta, originating at random cytosolic sites.
(MOV)

Movie S3 **Time-lapse recording of part of a C2 myotube expressing EB3-GFP (green) and GalT-mCherry (red), a Golgi complex marker.** The 12 o'clock sector of the middle nucleus is particularly active in EB3 puncta movement.
(MOV)

Movie S4 **Time-lapse recording of a C2 myoblast expressing EB3-GFP, before nocodazole treatment.**
(MOV)

Movie S5 **Time-lapse recording of a myoblast expressing EB3-GFP from Movie S4, after nocodazole washout.**
(MOV)

Movie S6 **Time-lapse recording of a C2 myotube expressing EB3-GFP before nocodazole treatment.**
(MOV)

Movie S7 **Time-lapse recording of a myotube expressing EB3-GFP from Movie S6, after nocodazole washout.**
(MOV)
