# Supplementary figures and images for: Who Needs Microtubules? Myogenic Reorganization of MTOC, Golgi Complex and ER Exit Sites Persists Despite Lack of Normal Microtubule Tracks
Source: PLoS One. 2011 Dec 27;6(12):e29057. doi: 10.1371/journal.pone.0029057 (PMC3246457; doi:10.1371/journal.pone.0029057)

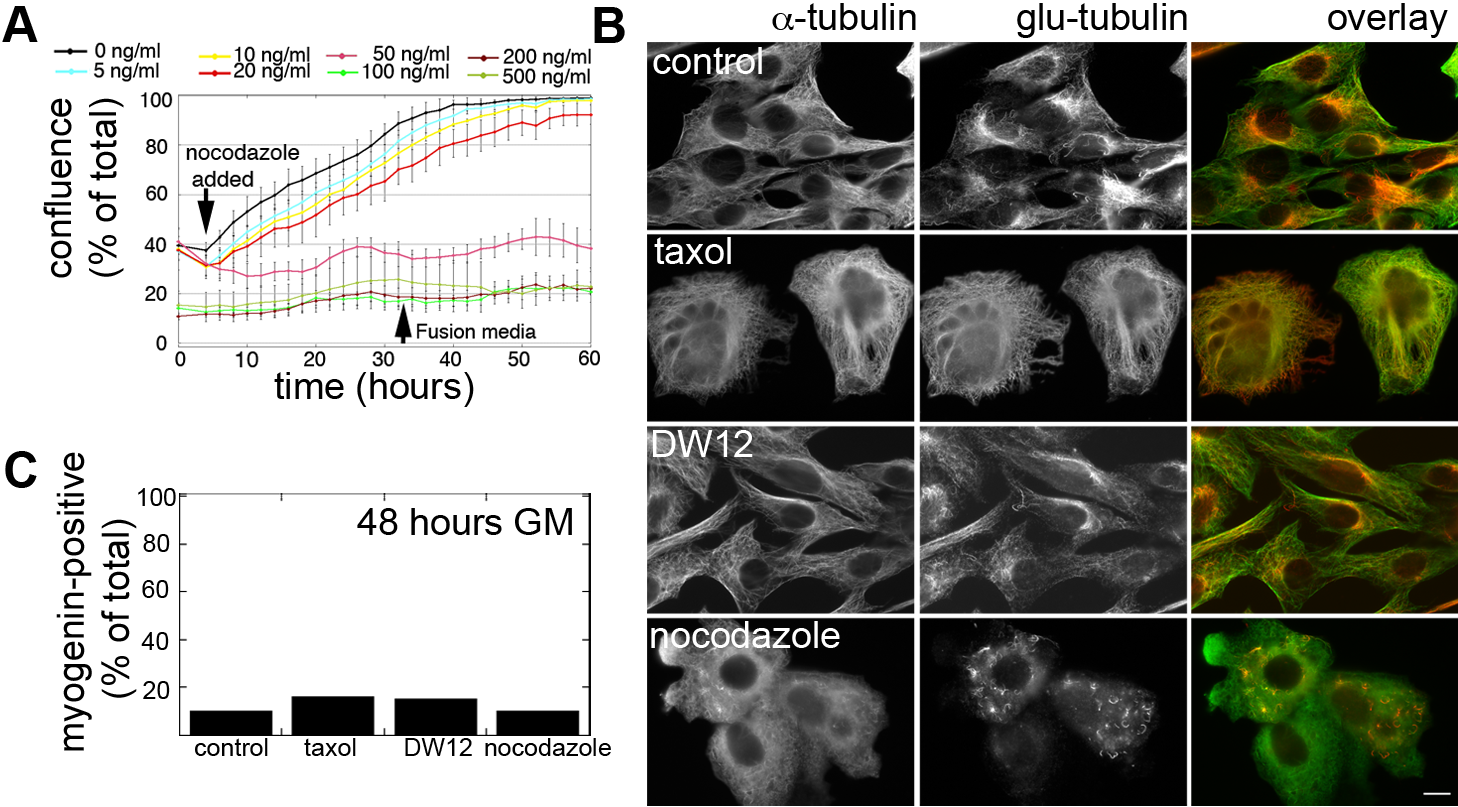

Supplement: Figure S1 — Effects of microtubule-affecting drugs on C2 cultures. (A) Optimization test for nocodazole concentration; cultures were imaged every 2 hours. Cell proliferation is arrested from 50 ng/ml on. (B) State of microtubules (α-tubulin, green) and microtubule stabilization (Glu-tubulin, red) before the switch to FM to initiate differentiation. (C) Myogenin expression in control and treated cultures after 2 days in GM, before the switch to FM to initiate differentiation. Wide-field images. Bar: 10 µm. (TIF) [file pone.0029057.s001.tif]

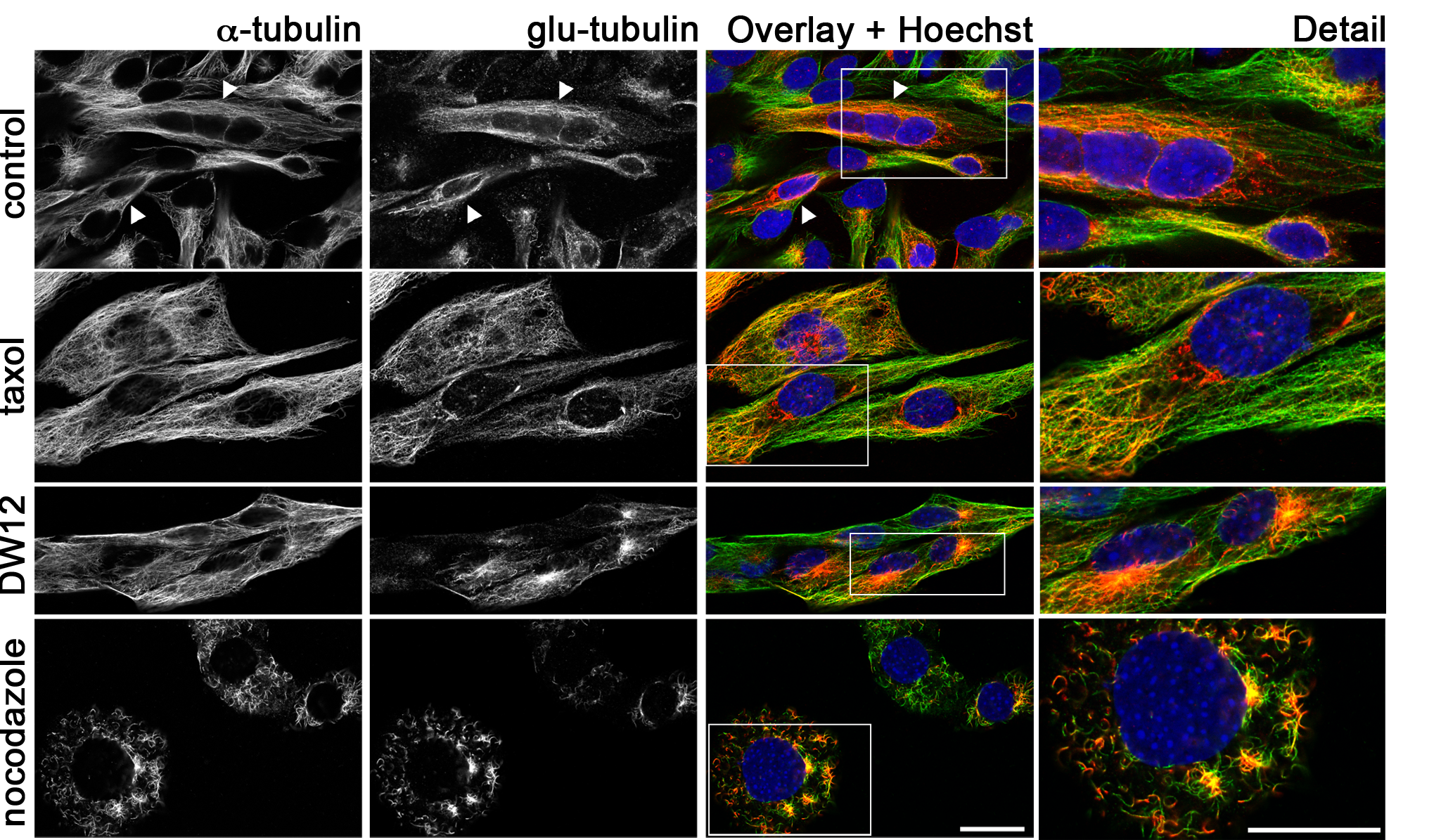

Supplement: Figure S2 — Cell shape and state of the microtubules in C2 cells differentiated in microtubule-disrupting drugs. Control cultures show many elongated cells and multinucleated myotubes with longitudinal microtubules (α-tubulin, green). Microtubule stabilization is reflected by Glu-tubulin staining (red, arrowheads), which increases in differentiated cells. In cultures treated with taxol (50 nM), the cells do not fuse but are large and elongated with high levels of Glu-tubulin. DW12 (100 nM) has no global effect on the microtubule network but, in most cells, increases Glu-tubulin next to the nucleus, and inhibits fusion. Nocodazole (200 ng/ml) prevents both elongation and fusion. Only very short microtubules are left, which partly stain for Glu-tubulin. Images are confocal z-stack projections. Outlined areas in the third column are shown enlarged in the last column. Nuclei are counterstained with Hoechst (blue). Bars: 25 µm. (TIF) [file pone.0029057.s002.tif]

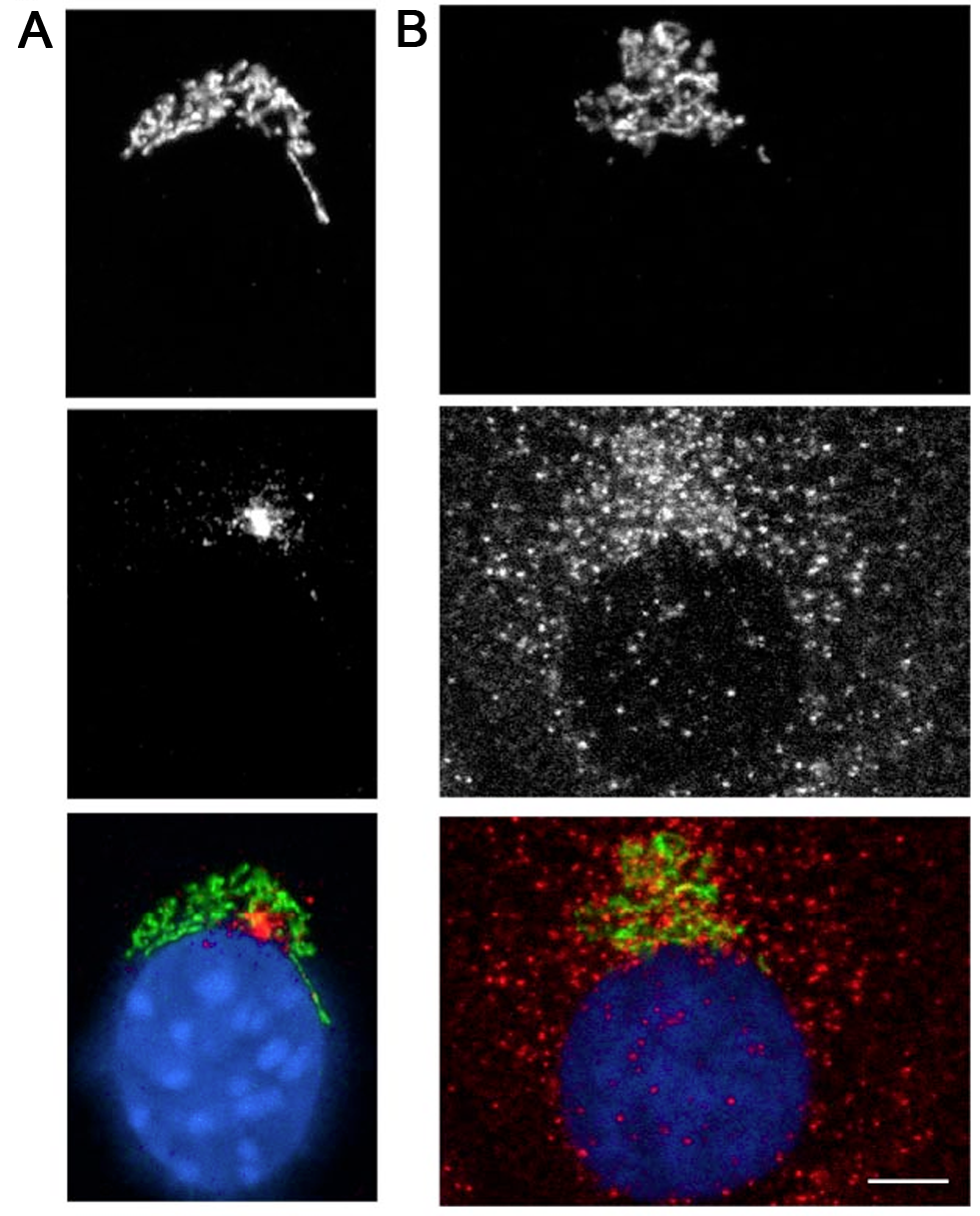

Supplement: Figure S3 — Organization of centrosomal proteins, Golgi complex and ERES in control undifferentiated cells. (A) Golgi complex identified by GM130 staining (top, green) and centrosome identified by pericentrin staining (middle, red). (B) GM130 staining (top, green) and ERES identified by Sec31 staining (middle, red). Bottom row: overlay with nuclear counterstain (blue). Wide-field images. Bar: 10 µm. (TIF) [file pone.0029057.s003.tif]

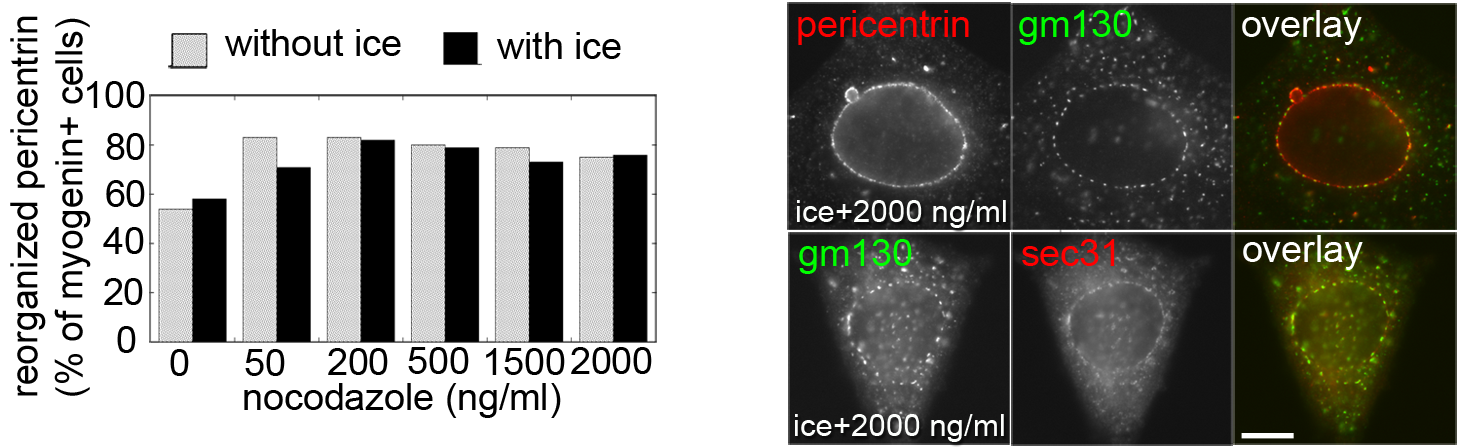

Supplement: Figure S4 — A large range of nocodazole concentrations and cold affect subcellular reorganization similarly. Cells with or without cold pretreatment were differentiated in FM with nocodazole for one day and myogenin-positive cells were assessed for the presence of perinuclear pericentrin belts. (A) All concentrations increased the fraction of cells with reorganized pericentrin. Cold pre-treatment did not have an additional effect. (B) Examples of cells with reorganized pericentrin, GM130 and Sec31 in cultures treated with cold and 2000 ng/ml nocodazole. Wide-field images. Bar: 10 µm. (TIF) [file pone.0029057.s004.tif]
